# Multidimensional assessment of time perception along the continuum of Alzheimer’s Disease and evidence of alterations in subjective cognitive decline

**DOI:** 10.1038/s41598-023-49222-x

**Published:** 2023-12-13

**Authors:** Alice Teghil, Maddalena Boccia, Antonella Di Vita, Giulia Zazzaro, Micaela Sepe Monti, Alessandro Trebbastoni, Giuseppina Talarico, Alessandra Campanelli, Giuseppe Bruno, Cecilia Guariglia, Carlo de Lena, Fabrizia D’Antonio

**Affiliations:** 1 Department of Psychology, Sapienza” University of Rome, Via Dei Marsi, 78, 00185 Rome, Italy; 2 Cognitive and Motor Rehabilitation and Neuroimaging Unit, IRCCS Fondazione Santa Lucia, Rome, Italy; 3 Department of Human Neuroscience, Sapienza University of Rome, Rome, Italy

**Keywords:** Neuroscience, Psychology

## Abstract

Timing alterations occur in Alzheimer’s disease (AD), even in early stages (mild cognitive impairment, MCI). Moreover, a stage named subjective cognitive decline (SCD), in which individuals perceive a change in cognitive performance not revealed by neuropsychological tests, has been identified as a preclinical phase of AD. However, no study to date has investigated different dimensions of time processing along the continuum from physiological to pathological aging, and whether timing alterations occur in SCD. Here a sample of participants with SCD, MCI, AD and healthy controls (HC) performed tasks assessing prospective duration estimation, production, reproduction, implicit temporal learning in conditions dependent from external cues (externally-cued learning, ECL) or independent from external cues (internally-based learning, IBL), retrospective duration estimation, the subjective experience of time and the temporal collocation of events. AD patients performed worse than HC and SCD in prospective timing, and in collocating events in time. The subjective experience of time did not differ between groups. Concerning temporal learning, AD performed worse in ECL than in IBL, whereas SCD performed worse in IBL than in ECL. SCD, MCI and AD patients all showed errors greater than HC in retrospective duration estimation. Results point to implicit temporal learning in externally-cued conditions and retrospective time estimation as possible early markers of cognitive decline.

## Introduction

The perception of time is a fundamental experience of human beings. It is an essential condition that allows persons to be in the world, act, behave and interact with the environment^1^. Karl Jaspers distinguished between time knowledge and time experience. The first refers to the measurable dimension of time, for which you can estimate a time interval, whereas the second refers to the inner feeling of the passage of time as an internal psychological phenomenon^2^. Both time dimensions are implicated in everyday life and they likely interact as complementary aspects. Mechanisms underpinning time perceptions are still not completely known. It has been hypothesized that time perception is based on an internal clock, described as a pacemaker-accumulator also including memory and decision stages^3^. In brief, the pacemaker would emit pulses that are accumulated in a counter: the number of pulses counted determines the perceived length of an interval.

This model was further expanded by the Attentional Gate Model^4^ which introduced an attentional gate between the pacemaker and the accumulator. When attention is devoted to time, the gate would open wider allowing the passage of more pulses to the accumulator. According to these models, working memory, executive functions as well as attention are fundamental to time perception^5–7^.

Time perception, meant as time knowledge, has been traditionally investigated using prospective timing paradigms, in which participants are informed that a temporal judgment will be required, or by means of retrospective paradigms, in which participants are not previously informed that they will be asked to judge a duration^8^. Most commonly used prospective timing paradigms include verbal estimation (requiring a verbal estimate of presented durations), production (in which a duration specified in temporal units has to be produced) and reproduction (requiring participants to perform a response to reproduce the length of previously presented time intervals)^9^. Retrospective time perception tasks, instead, are often limited to a single trial and require participants to verbally estimate a time interval filled with other (non-temporal) tasks^10^. Whereas prospective timing depends on different cognitive processes, such as attention and working memory, retrospective timing mainly depends on the ability to remember contextual information associated with the time period and to retrieve it after the duration has elapsed^10^.

The subjective experience of time has also been investigated using different methods, including questionnaires assessing the perceived passage of time and the temporal collocation of events^11–13^, and tasks requiring to estimate the date of public events^14^.

In Alzheimer’s disease (AD), even in early stages (i.e. Mild cognitive impairment, MCI), alterations in time perception occur. Patients lose track of time, orientation in time and the capacity to collocate events in correct temporal order^15^.

Time perception has been investigated in AD using different tasks. Using prospective verbal estimation in the range of multiple seconds, AD patients have been shown to mainly overestimate durations^6^ and to be less accurate^16–18^ than healthy controls (HC). Partially at odds with the abovementioned reports of overestimation in verbal estimation paradigms^6^, multiple-second durations have also been reported to be both underestimated and under-reproduced by AD patients in prospective paradigms^19,20^. Also, the same durations were underestimated when judged in a retrospective paradigm^20^.

Few studies have investigated time perception in patients with MCI, with some reporting no differences with HC in prospective verbal estimation^18,21^ or production^21^ of multiple seconds durations. Concerning the range of milliseconds to a few seconds, instead, no difference between MCI and HC has been reported using time bisection and finger-tapping^22^. Nonetheless, working-memory deficits have been reported to specifically affect performance of MCI patients in this range when multiple durations have to be reproduced^23^. Concerning retrospective time processing, instead, the only study specifically performed on MCI patients reported no differences with HC^21^. Overall, the findings from these studies using different paradigms and tasks, and assessing non-systematically variable duration ranges in different populations, are not conclusive about alterations of time perception in AD and MCI (see^10^ for a meta-analysis pointing out to these and other methodological issues).

Concerning the subjective experience of time, AD patients have been found to show larger errors in dating past events compared to HC^14^. Moreover, difficulties have been reported in MCI in perceiving relations between the past, present, and future^15^, together with the feeling of time passing more slowly^21^. Again, this sparse body of research does not allow to draw definite conclusions on possible alterations of subjective time in AD and its prodromal stages.

Before overt cognitive impairment occurs, a stage has been identified named Subjective cognitive decline (SCD), in which individuals perceive a change in their cognitive performance not revealed by cognitive tests. This stage can be considered as a preclinical phase preceding dementia onset, since AD neuropathological process starts years before overt dementia onset^24^. However, whether alterations in duration processing and time experience occur in SCD has not been investigated to date. Overall, no studies have investigated all time dimensions systematically, along the continuum from physiological to pathological aging, i.e. SCD, MCI and AD.

The main aim of this study was to provide a comprehensive investigation of different dimensions of time processing. Thus, patients with AD, MCI, individuals with SCD and healthy elderlies performed a battery of tasks investigating different dimensions of time and duration processing. In line with previous studies^6,16–20^, explicit prospective timing was assessed using duration estimation, production and reproduction paradigms. Importantly, dissociations have been reported in healthy aging between temporal prediction tasks, assessing the representation of time implicitly, and tasks requiring overt duration judgements, that assess the representation of time explicitly^25,26^. Thus, here we also investigated implicit time processing along the AD continuum, using a novel task assessing implicit temporal learning. More in detail, based on evidence that different neurocognitive mechanisms mediate timing when events and responses are timed independently from variations in perceptual features of the stimuli or other external cues (internally-based timing, IBT), and when they are timed based on exogenous sensory signals (externally-cued timing, ECT)^27–30^, temporal learning was assessed both in an internally-based (IBL) and an externally-cued condition (ECL). Based on previous literature, suggesting that retrospective timing may be impaired in AD and MCI^10^, the estimation of duration in retrospect was also assessed. Finally, we further performed a systematic assessment of the feeling of the passage of time (including both the subjective time experience and the collocation of events in time) along the AD continuum.

We hypothesized that specific alterations may characterize preclinical and prodromal stages of AD, including SCD. First, we hypothesized that explicit and implicit timing could be differently affected along the progression from healthy aging to AD. Moreover, based on evidence that IBT and ECT processes depend on different neural mechanisms^27^ and are affected by different patterns of brain damage^30^, we hypothesized that IBL and ECL could be also differentially affected along the continuum of AD. Finally, we hypothesized that alterations in retrospective time processing may be observed also in individuals with SCD, who often perceive a reduction in their memory skills^31^.

## Methods

### Participants

16 patients with a diagnosis of Subjective Cognitive Decline (SCD), 17 with a diagnosis of Mild Cognitive Impairment (MCI), 13 with a diagnosis of Alzheimer’s Disease (AD) and 17 healthy controls (HC) took part in the study. This sample size was in line with previous studies assessing time perception in similar samples of patients^19,20,22^. The diagnosis of probable AD and MCI was made according to the clinical criteria from the National Institute on Aging-Alzheimer’s Association workgroups (NIA-AA)^32,33^. The diagnosis of SCD was based on subjective cognitive decline criteria^34^. We included as SCD individuals who spontaneously referred to the memory clinic seeking for medical help and who were worried about their cognitive efficiency^31^*.* Patients underwent a physical and neurological assessment, standard laboratory tests, serum vitamin B12, folate, and thyroid hormone assays as well as a neuropsychological evaluation. Patients were included if they were between 55 and 86 years of age. Patients were excluded if they had secondary causes of dementia, degenerative dementia other than AD, or vascular dementia diagnosed according to the National Institute of Neurological Disorders and Stroke and Association Internationale pour la Recherche et l’Enseignement en Neurosciences (NINDS-AIREN) criteria^35^. Patients and healthy subjects were excluded if they had psychiatric comorbidities, if they had had repeated head trauma, protracted loss of consciousness following head trauma or severe central nervous system infections within the last 5 years, or if they had a history of cerebrovascular disease (i.e., stroke, transient ischemic attacks, cerebral hemorrhage). Patients who were taking antipsychotic drugs and benzodiazepines were excluded while those taking anticholinesterase inhibitors were not. Demographic information on participants is reported in Table 1. Groups were matched for age (F(3,59) = 2.684, p = 0.055), education (F(3,59) = 1.88, p = 0.143), and gender (χ2 = 0.509, p = 0.917) but were significantly different on MMSE score (F (3,59) = 28.045, *p* < 0.001), with HC and SCD being significantly different from AD and MCI patients (all ps < 0.001, Bonferroni corrected). The study was designed in accordance with the principles of the Declaration of Helsinki and was approved by the Ethical Committee of Sapienza University of Rome (Prot. 5179, 10/10/2018). Written informed consent was obtained from all individual participants included in the study.

**Table 1:** Demographic information on participants.

Group	N	F/M	Age (SD)	Education (SD)	MMSE (SD)
HC	17	10/7	74.12 (4.23)	12.94 (4.71)	28.65 (1.27)
SCD	16	8/8	73.56 (3.83)	12.37 (4.44)	28.56 (1.26)
MCI	17	9/8	73.94 (6.48)	12.18 (4.71)	24.47 (2.50)
AD	13	8/5	78.31 (5.19)	9.23 (4.13)	22.85 (3.18)

Means and standard deviations for age, education and raw MMSE score are reported. HC = healthy controls; SCD = Subjective Cognitive Decline patients; MCI = Mild Cognitive Impairment patients; AD = Alzheimer’s Disease patients; F = females; M = males.

### General procedure

The Bouncy ball task, the duration estimation, production and reproduction tasks and the Newscast task (see below) were developed as computerized paradigms. All computerized tasks were developed and presented using E-Prime 3.0 (Psychology Software Tools, Pittsburgh, PA) on a Lenovo V330-15IKB laptop. The retrospective duration estimation task and the assessment of the subjective experience of time were administered in a paper-and-pencil form.

Participants performed all the experimental tasks in a single session, lasting ~ 2 h. As a part of the standard diagnostic procedure, in a separate session participants of the SCD, MCI and AD groups also underwent a neuropsychological examination, including the Rey Auditory Verbal Learning Test^36^, Rey-Osterrieth Complex Figure Test^36^, Copy of Rey-Osterrieth’s Complex Fig.^36^, Corsi Block-Tapping Test^37^, Digit Span Test^38^, Babcock Story Recall Test^38^, Visual Search^38^, Trail Making Test^38^, Phonemic Verbal Fluency task^39^, Semantic Verbal Fluency task^39^, Boston Naming Test^40^, Frontal Assessment Battery^41^.

### Internally-based and externally-cued temporal learning: the Bouncy ball task

We assessed temporal learning in internally-based (IBL) and externally-cued (ECL) conditions using an adaptation of the temporal learning paradigm previously developed by our group in the context of a study in healthy participants^42^. This task was found to be effective in fostering temporal learning, and performance in the two conditions (IBL and ECL) has been shown to be differently affected by individual variability in high-level cognitive functioning^42^, in line with evidence supporting the dissociation between internally-based and externally-cued timing mechanisms^27–30^.

As in our previous study, the Bouncy ball task was aimed to test the learning of a timed response based on the repeated exposure to a fixed duration (internally-based timing) or to a regular visual pattern (externally-cued timing). At difference with Teghil et al.^42^, here we choose to present a stimulus more closely resembling a physical object. This allowed us to increase face ecological validity, and to ensure that also cognitively impaired participants (e.g. AD patients) may comply with task instructions.

**Figure 1:**
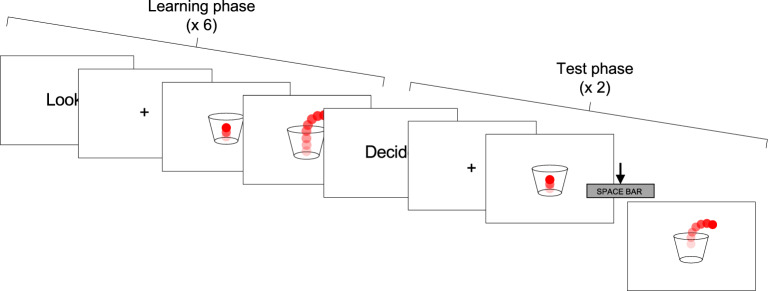
A trial of the temporal learning task. The task had the same structure in the IBL and ECL conditions. In the IBL condition, the bouncy ball bounced for two different numbers of times within the six repetitions of the observation phase of a trial, though total bouncing time before exiting the cup was the same within the trial. In the ECL condition, the bouncy ball bounced for two different time intervals within the six repetitions of the observation phase of a trial, though its total number of bounces was the same within the trial. At the beginning of the learning phase, the instruction “Look” was presented for 1000 ms, followed by a fixation cross for 1000 ms. Then, the clip showing the bouncing stimulus was presented for six repetitions. In the test phase of each trial, the instruction “Decide” was displayed for 2000 ms, followed by a fixation cross for 1000 ms. Then the test clip was presented, and participants were instructed to press the spacebar key to make the bouncy ball exit the cup. Two responses were required for each trial. After the response was provided, the words “Well done!” were presented for 3000 ms. The word “Rest” was then presented for 1000 ms before the starting of a new trial.

In the Bouncy ball task, participants were presented with brief videoclips, showing a red ball bouncing into a transparent cup, and eventually bouncing out of the cup at a given moment (Fig. 1). In the learning phase of each trial, participants were asked to observe the moving stimulus for six repetitions. Then, in the test phase, they were shown the same moving stimulus, and were instructed to press the spacebar when they thought the ball should bounce out of the cup (the spacebar press actually caused the ball to bounce out). In line with our previous study^42^, there were two task conditions: in the ECL condition, the number of times the ball bounced defined a duration (thus, durations were specified by external cues), whereas in the IBL condition, the number of times the ball bounced varied for a given duration (therefore, an internal representation of duration had to be built independently from external cues). More in detail, in the IBL condition, the total bouncing time (i.e. the duration) of the stimulus was the same in each of the six repetitions of a given trial; the number of times the stimulus bounced, however, varied in different repetitions of the same trial. Conversely, in the ECL condition, the time duration for which the ball bounced before exiting the cup was variable within the six repetitions of a given trial, although the number of times it bounced was fixed. In other words, in each repetition of a given trial in the IBL condition, the ball always exited the cup after a fixed time interval; the number of times the ball bounced before exiting the cup, instead, took two different values within each trial (one in three repetitions, and a different one in the other three), thus the total number of bounces differed between one half of the repetitions and the other. In the ECL condition, instead, the bouncy ball always bounced a fixed number of times in all the six repetitions of a given trial; however, since the bouncing speed varied between one half of repetitions of a trial and the other, the total bouncing time took two different values within a single trial. Thus, in the IBL condition, the decision about when making the bouncy ball exit the cup depended on the formation of a fixed temporal referent despite perceptual variations in the stimulus. Instead, in the ECL condition, the decision depended on the development on an external referent, since participants had to rely entirely on visual information (i.e. the number of bounces).

The conditions were presented in two separate blocks, each including six trials. The presentation order of the two blocks was balanced within each group. Each trial involved six presentations of the bouncing ball (learning phase) and required participants to provide two responses (test phase). In each trial the two parameters (number of bounces/duration) in the six repetitions followed a fixed randomized order. The bouncing speed of the bouncy ball in the test phase was fixed, but always different from that in the six presentations of the learning phase, ensuring that participants had to base their decision on acquired information about the bouncing duration (IBL) and number of bounces (ECL) of the ball to correctly perform the task. No reference to time or duration was made when giving task instructions.

Bouncing time, number of bounces, and speed (frames per second, FPS) parameters in each Condition and trial are reported in Supplementary materials (Table S1). The IBL and ECL conditions were matched for mean bouncing duration (IBL: M = 4716.667, SD = 1385.859; ECL: M = 3599.999, SD = 1134.225; U = 96, p = 0.178), mean number of bounces (IBL: M = 7.000, SD = 2.954; ECL: M = 6.333, SD = 2.146; U = 80, p = 0.671) and mean speed (IBL: M = 23.333, SD = 7.177; ECL: M = 30.833, SD = 14.899; U = 52, p = 0.266).

### Prospective duration estimation, production and reproduction tasks

The same five durations (2000, 3100, 4700, 5500, 6300 ms) were tested in each task; each duration was presented 5 times, for a total number of 25 trials in each task. All stimuli were presented on a white background. The presentation order of trials within the tasks was randomized. Tasks were also presented in a pseudo-randomized order, with the constrain that the duration production task was always presented last, in order to ensure that participants could not use explicit knowledge of the target durations to perform the duration estimation and reproduction tasks.

#### Duration estimation task

**Figure 2:**
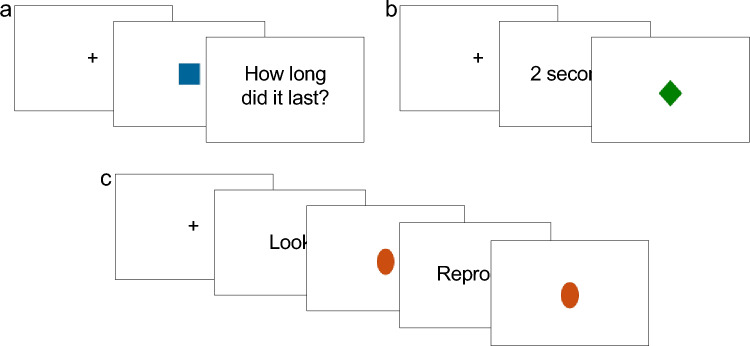
Schematic representation of task events in the duration estimation (DE), duration production (DP) and duration reproduction (DR) tasks. (**a)** DE task. Each trial started with a fixation cross, displayed until the spacebar key was pressed. The spacebar key press triggered the presentation of a shape, that was displayed for a target amount of time. Then, a question asking “How long did it last?” appeared on screen until the participant provided a verbal response. The spacebar key was then pressed to start the next trial. (**b)** DP task. Each trial started with a fixation cross, displayed until the spacebar key was pressed. The spacebar key press triggered the presentation of a written label, indicating the duration to be produced. The label was presented until the spacebar key was pressed (or for a maximum of 10,000 ms, after which the task proceeded automatically), and this pression triggered the presentation of a shape. The shape was displayed until participants pressed the spacebar key again, ending the production phase. After a 1500 ms delay, the next trial started automatically. (**c)** DR task. Each trial started with a fixation cross, displayed until the spacebar key was pressed. The spacebar key press triggered the presentation of the instruction “Look” for 1000 ms, after which a shape was displayed for a target duration (encoding phase). The instruction “Reproduce” was then presented for 2000 ms, after which the shape was displayed again, marking the beginning of the reproduction phase. The shape was displayed until participants pressed the spacebar key again, ending the reproduction phase, or for a maximum of 30,000 ms, after which a warning appeared and the trial was deemed as missing. After a 1500 ms delay, the next trial started automatically.

The duration estimation (DE) task (Fig. 2a) required to estimate the presentation time of a simple shape (a blue square, RGB: 8/101/153, 3.9 × 3.9 cm). The shape was presented at the center of screen. The presentation of the stimulus was triggered by a spacebar press. After the presentation duration has elapsed, a question appeared on screen asking “How long did it last?”, and participants were instructed to verbally provide an estimate of the stimulus duration. The experimenter took note of estimated durations.

#### Duration production task

In the duration production (DP) task (Fig. 2b), participants had to produce time intervals equivalent to previously indicated durations. Durations to be produced were presented on screen as written labels (e.g. “2 s”, “3.1 s”). Participants were asked to press the spacebar to start the production phase, and to make a second spacebar press to terminate the produced duration. The first spacebar press triggered the presentation of a simple shape (a green rhomb, RGB: 9/128/2, 3.96 × 3.53 cm), that appeared at the center of screen immediately after the first spacebar press, and disappeared when the participants pressed the spacebar key the second time. If the second bar press did not occur within 30,000 ms, the shape disappeared, and a warning was displayed.

#### Duration reproduction task

In the duration reproduction (DR) task (Fig. 2c) participants reproduced the presentation time of an orange oval (RGB: 203/78/17, 5.06 × 3.43 cm). The starting of each trial was triggered by a spacebar press, after which the instruction “Look” was displayed for 1000 ms. The shape was then presented for one of the target intervals (encoding phase). Right after, the instruction “Reproduce” was presented for 2000 ms, and the shape was presented again. Participants were instructed to press the spacebar key to make the shape disappear when they thought it had been presented for the same time as the encoding phase. If a spacebar press in the reproduction phase did not occur within 30,000 ms, the shape disappeared and a warning was displayed.

### Retrospective duration estimation task

After completion of the Bouncy ball task, DE, DP and DR tasks, participants were asked to provide a verbal estimate of the duration of the whole experimental session up to that point. Estimates were noted down by the experimenter.

### Duration estimation in the minute range and estimation of the date of public events: the Newscast task

A novel task was developed to assess the ability to estimate the date of public events; the same task was also used to assess the estimation of durations in the minute range. In the Newscast task (NT) 9 auditory clips were presented, taken from publicly available Italian television newscasts. Each clip focused on a specific public event happened within one of three time periods: this year, within the last 5 years, more than 5 years ago (three events for each period). Periods were chosen based on evidence that the recollection of recent and remote memories involves at least partially separate brain networks^43,44^. Within each period, events were drawn from three broadly defined thematic categories (news stories, politics and entertainment news).

Participants were asked to listen to the clips and, after each clip, to answer the following questions: (a) To which category was the clip drawn from? (Multiple choice question; possible answers were: news stories, politics, entertainment news); (b) Did you know about the event described in the clip? (Yes/No); (c) When do you think this event had taken place?; (d) How long do you think this clip has lasted? (Open-ended questions).

Duration of the clips ranged between 43 and 204 s (M = 90.111, SD = 40.565). In order to investigate possible differences in the estimation of durations in the minute range, clips were balanced to span across different duration ranges. Specifically, within each time period, the length of each of the three clips was (1) below 1 min (M = 50.5 s, SD = 5.577, “short” duration range), (2) between 1 and 2 min (M = 85.17 s, SD = 10.572, “medium” duration range), and (3) above 2 min (M = 134.67 s, SD = 34.022, “long” duration range).

The specific topic, duration and airing date of each clip is reported in Supplementary Materials (Table S2). Since data were collected between 2017 and 2019, partially different clips were used during data collection (see Table S2). The two sets of clips used were not significantly different for duration (2017: M = 95.333, SD = 49.409; 2018: M = 84.889, SD = 10.510; U = 38, p = 0.863). The order of presentation of clips was randomized.

### Assessment of the subjective experience of time

Participants completed a self-report questionnaire assessing their subjective experience of time. Items were selected from existing questionnaires assessing the experience of time and time awareness^11–13^. Specifically, items assessing the Personal experience of present (e.g. “How fast does usually time pass for you?”, two items) and past (“How fast did the previous week pass for you?”, 8 items), the feeling of Time pressure (e.g. “I haven't enough time to complete my tasks”, 5 items) and of Time expansion (e.g. “My time seems empty”, 5 items) were selected from the Subjective Time Questionnaire^11,12^. Items assessing the Experience of recent life changes (e.g. “The past two years have been a time filled with many new experiences”, 4 items) and the experience that one often underestimates the ages of events (forward telescoping) (e.g. “When I try to remember the date of some event, I often come up with a time that is not as long ago as the true time”, 3 items) were selected from the Study of the Experience of Time questionnaire^13^. All items were rated on a five-point scale. The complete list of items included in the questionnaire administered in the present study is reported in Supplementary materials (Supplementary file S1).

### Statistical analyses

Since preliminary analyses showed that data did not generally meet the assumptions of parametric analyses, robust or non-parametric statistical methods were used in the following analyses.

### Bouncy ball task

Average absolute error scores were calculated separately for the IBL and ECL conditions using the formula|(Reproduced duration – Target duration)/Target duration|). It is important to point out that whereas in the IBL condition the target duration was equal to the fixed bouncing time of the stimulus in that specific trial, target duration in the ECL condition corresponded to time elapsed between the starting of the test phase, and the moment in which participants should make the bouncy ball exit the cup based on the number of times it had bounced (see task description). We focused analyses on error measures based on reproduced durations in order to allow the comparison between the IBT and ECT condition (see also^42^ for a similar approach).

Trials in which the reproduced duration was below 300 ms or above/below 2 standard deviations from the whole sample’s mean for that specific duration in each specific condition were excluded from further analyses. These represented 14.55% of the total number of trials.

In order to compare absolute error scores in the IBL and ECL conditions between the four groups (healthy controls [HC], SCD participants [SCD], MCI patients [MCI] and Alzheimer’s Disease patients [AD]), we performed a Welch–James test with Approximate Degrees of Freedom (Welch ADF), that allows to deal with heterogeneous distributions and non-normally distributed data in mixed-factorial designs. Bootstrapping was used to calculate p values for main effects and interactions. The Welch–James test was performed with condition (IBL, ECL) as within-subjects factor, and Group (HC, SCD, MCI, AD) as between-subjects factor. The analysis was performed using the welchADF package^45^ for R. The origin of the interaction effect was specified using Wilcoxon’s tests (see^42,46,47^ for similar procedures); alpha level was set at 0.017 for this analysis, applying Bonferroni’s correction for multiple comparisons.

### Prospective duration estimation, production and reproduction tasks

Average absolute error scores in each task were calculated using the formula|(Estimated/produced/reproduced duration – Target duration)/Target duration|). Concerning the duration production and reproduction tasks, trials in which the produced/reproduced duration was below 300 ms or above/below 2 standard deviations from the whole sample’s mean for the specific duration in the task were excluded from further analyses (4.190% of trials in the production and 7.111% of trials in the reproduction task). The ratio of the estimated/produced/reproduced to the target duration was also analyzed to assess the directionality of errors between groups; results are reported in Supplementary materials (Supplementary file S2).

Based on previous evidence that patients with amnestic MCI appear to rely more strongly on prior experience than healthy controls when reproducing multiple durations^23^, we further calculated the slope of the linear regressions of the target duration on the estimated, produced or reproduced duration in each group as a measure of central tendency effects. A single observation from the AD group was removed from this analysis in the duration estimation task, since this value was found to be an extreme outlier (more than 3rd quartile + 1.5*interquartile range).

We first compared error scores between groups for each task separately, performing three Kruskal–Wallis tests. When a significant difference was identified, paired comparisons were performed between groups using Mann–Whitney tests (alpha level was set at 0.0083 for this analysis, applying Bonferroni’s correction for multiple comparisons). Central tendency was also compared between groups for each task separately, performing three Kruskal–Wallis tests. Again, Mann–Whitney tests were performed for post-hoc analyses, setting alpha level at the same valued mentioned above.

### Retrospective duration estimation task

Average absolute error scores were calculated using the formula|(Estimated duration – Target duration)/Target duration|). Scores were compared between groups (HC, SCD, MCI, AD) using a Kruskal–Wallis test. Paired comparisons between groups were then performed using Mann–Whitney tests (alpha level was set at 0.0083 for this analysis, applying Bonferroni correction). The ratio of the estimated to the target duration was also analyzed; results are reported in Supplementary materials (Supplementary file S2).

### Newscast task

#### Duration estimation in the minute range

Since there was no difference in duration between the two sets of clips (see task description), the two sets were merged in following analyses. Average absolute standardized error scores were calculated using the formula|(Estimated duration – Target duration)/Target duration|). To assess possible differences as a function of the duration range, independently from the group (i.e. testing the main effect of Duration range), a Friedman test was performed on ASE with the factor Duration range (short, medium, long). A Kruskal–Wallis test was also performed on ASE to assess the main effect of Group (HC, SCD, MCI, AD), independently from the duration range. Finally, the interaction between factors Group and Duration range was also assessed adopting a non-parametric approach: we first calculated differences between mean ASE scores in each group for each pairing of levels of the Duration range factor (i.e. mean ASE was calculated within each group for differences between the short and medium condition, the short and long condition, and the medium and long condition). Then, groups were compared on such differences using Kruskal–Wallis tests. Wherever Kruskal–Wallis tests showed that differences between the levels of the repeated-measures factor did indeed differ significantly between groups, groups’ means were compared using Mann–Whitney tests (alpha was set at 0.0083, applying Bonferroni correction). The same analysis was further performed on the ratio of the estimated to the target duration; results are reported in Supplementary materials (Supplementary file S2).

#### Estimation of the date of public events

Only clips for which participants reported to have known about the corresponding event were included in this analysis. Answers were scored as correct if the date of the clip was collocated within the expected time period (this year [TY], within the last 5 years [< 5], more than 5 years ago [> 5]).

Adopting the same approach described above to assess duration estimation in the minute range, we first tested the main effect of Time period (TY, < 5, > 5), performing a Friedmann Test on accuracy in collocating the clips in time, independently from Group. Wilcoxon tests for paired samples were then used to perform three paired comparisons between levels of the factor Time period (alpha was set as 0.017, Bonferroni corrected). The main effect of Group was assessed performing a Kruskal–Wallis test on accuracy; pairwise comparisons were then performed using Mann–Whitney tests (alpha = 0.0083). The interaction between factors Group and Time period was assessed using a non-parametric approach, again, first calculating differences between mean accuracy in each group for each pairing of levels of the Time period factor (i.e. mean accuracy was calculated within each group for differences between the TY and < 5 condition, the TY and > 5 condition, and the < 5 and > 5 condition). Then, groups were compared on such differences using Kruskal–Wallis tests.

#### Backward and forward telescoping

Further analyses were performed to assess differences due to Group and Time period in backward and forward telescoping effects, i.e. the erroneous attribution of events to respectively earlier and later dates^14,48^. Each answer was categorized as “backward”, “forward”, or “correct” (responses for which the year was correctly identified were considered correct also if participants were not able to provide the exact date). We then calculated the proportion of backward, forward and correct responses for each period, and computed, for each participant in each period, an index of telescoping (telescoping index, TI) using the following formula: proportion of correct responses – proportion of backward responses + proportion of forward responses. This allowed us to quantify the individual tendency to attribute events to earlier or later dates. The main effect of Time period was then assessed performing a Friedman test on TI; post-hoc comparisons were performed using three paired Wilcoxon tests (alpha set at 0.017). The main effect of Group was tested performing Kruskal–Wallis tests on TI. Finally, as in the previous analysis, the interaction between Group and Time period was assessed non-parametrically calculating differences between groups in differences between levels of the factor Time period.

### Subjective experience of time

Kruskal–Wallis tests were performed to assess differences between groups on the factors Personal experience of present, Personal experience of past, Time pressure, Time expansion, Experience of recent life changes and Forward telescoping.

### Correlation with neuropsychological tests

Two-tailed Spearman’s correlations were performed between scores on neuropsychological tests (MMSE, Rey Auditory Verbal Learning Test, Rey-Osterrieth Complex Figure Test, Babcock Story Recall Test, Digit Span Test, Corsi Block-Tapping Test, Copy of Rey-Osterrieth’s Complex Figure, Visual Search, Trail Making Test, Phonemic Verbal Fluency task, Semantic Verbal Fluency task, Boston Naming Test, Frontal Assessment Battery) and performance in the experimental tasks (Bouncy ball task, duration estimation, production and reproduction tasks, retrospective duration estimation task, duration estimation in the minute range, estimation of the date of public events, Personal experience of present, Personal experience of past, Time pressure, Time expansion, Experience of recent life changes and Forward telescoping). Results were corrected for multiple comparisons applying Bonferroni’s correction, setting alpha level at 0.0013.

## Results

### IBL and ECL

**Figure 3:**
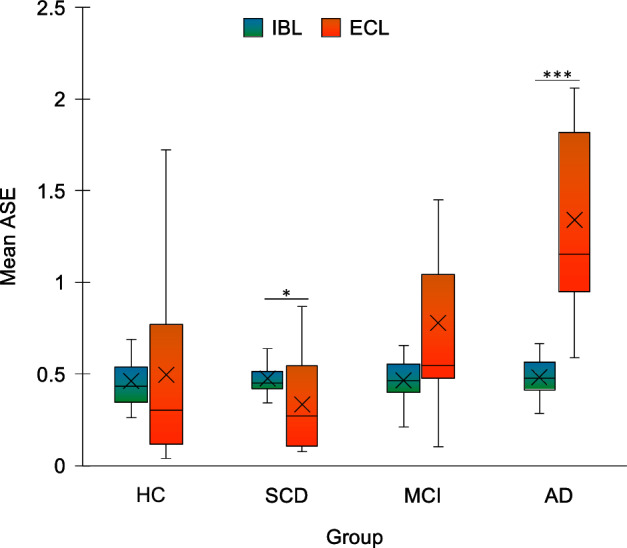
Results of the Bouncy ball task. For each group, mean absolute standardized error (ASE) is plotted for each condition of the task. IBL = Internally-based learning condition; ECL = Externally-cued learning condition; HC = healthy controls; SCD = Subjective Cognitive Decline; MCI = Mild Cognitive Impairment; AD = Alzheimer’s Disease. *p < 0.05 ***p < 0.001. Error bars represent standard errors.

We found a significant effect of Condition (WJ_1,25.17_ = 10.771, p = 0.003), with greater absolute standardized errors (ASE) in ECL (M = 0.707, SD = 0.578) than IBL (M = 0.472, SD = 0.124). The effect of Group was significant (WJ3,19.75 = 11.634, p = 0.001): ASE were higher in AD (M = 0.913; SD = 0.554) compared to HC (M = 0.480, SD = 0.349), SCD (M = 0.407, SD = 0.200) and MCI (M = 0.624, SD = 0.435), and also higher in MCI compared to SCD (HC vs. AD: U = 25, *p* < 0.001; SCD vs. MCI: U = 59, p = 0.006; SCD vs. AD: U = 5, *p* < 0.001; MCI vs. AD: U = 37, p = 0.002). The Group × Condition interaction was significant (WJ_1,19.26_ = 9.202, p = 0.006, bootstrap critical value for Family-Wise Error Rate control = 9.849): AD performed worse in the ECL than in the IBL condition (IBL: M = 0.484, SD = 0.108; ECL: M = 1.341, SD = 0.378; Z = -3.18, *p* < 0.001), whereas SCD individuals performing worse in the IBL than in the ECL condition (IBL: M = 0.477, SD = 0.109; ECL: M = 0.336, SD = 0.245; Z = -2.379, p = 0.017) (Fig. 3). Exploring the interaction effect the other way around (i.e. specifying the origin of the interaction using Mann–Whitney’s tests to compare groups, with alpha level set at 0.0125 applying Bonferroni correction), results showed that, in the ECL condition only, AD patients performed worse than CT (U = 20, *p* < 0.001) and SCD participants (U = 4, *p* < 0.001).

### Prospective duration estimation, production and reproduction

**Figure 4:**
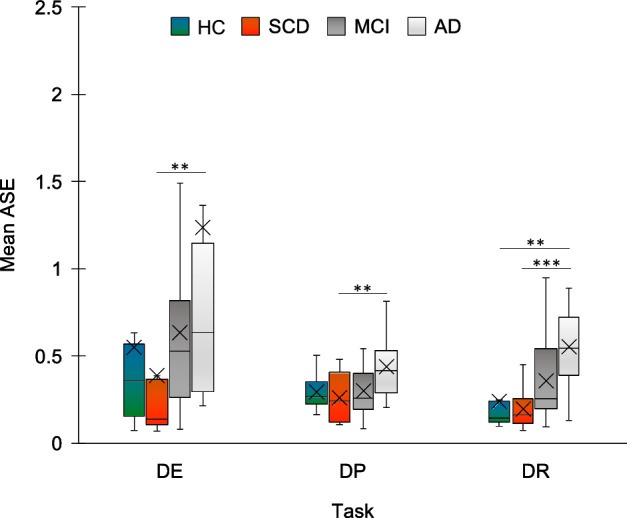
Results of the duration estimation, production and reproduction tasks. Mean absolute standardized error (ASE) is plotted according to each task and group. DE = duration estimation; DP = duration production; DR = duration reproduction; HC = healthy controls; SCD = Subjective Cognitive Decline; MCI = Mild Cognitive Impairment; AD = Alzheimer’s Disease. **p < 0.01 ***p < 0.001. Error bars represent standard errors.

There was a significant difference in ASE between groups in duration estimation (DE) (H_3_ = 10.401, p = 0.015) production (DP) (H_3_ = 10.223, p = 0.017), and reproduction (DR) (H_3_ = 20.068, *p* < 0.001). Post-hoc comparisons showed that in DE, SCD (M = 0.387, SD = 0.533) performed better than AD (M = 1.236, SD = 1.998) (U = 41, p = 0.006). SCD performed better than AD also in DP (SCD: M = 0.260, SD = 0.145; AD: M = 0.437, SD = 0.164: U = 42, p = 0.007). In DR, both HC and SCD performed better than AD (HC: M = 0.241, SD = 0.201; SCD: M = 0.197, SD = 0.113; AD: M = 0.551, SD = 0.225; HC vs. AD: U = 32, p = 0.001; SCD vs. AD: U = 18.5, *p* < 0.001) (Fig. 4). No other difference survived correction for multiple comparisons.

**Figure 5:**
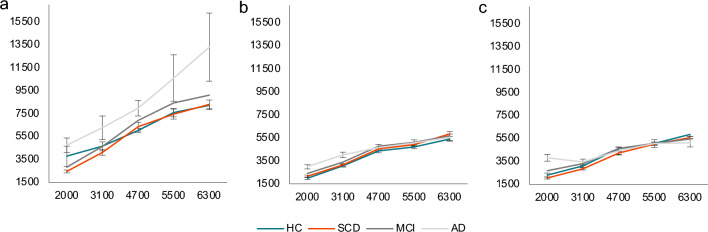
Estimated (panel a), produced (panel b) and reproduced (panel c) durations as a function of target durations for each group. HC = healthy controls; SCD = Subjective Cognitive Decline; MCI = Mild Cognitive Impairment; AD = Alzheimer’s Disease. Error bars represent standard errors.

Estimated, produced and reproduced durations as a function of target duration in the four groups are shown in Fig. 5. Concerning central tendency effects, we found a significant difference between groups in DR (H_3_ = 14.078, p = 0.003), but not in DE (H_3_ = 3.858, p = 0.277) nor DP (H_3_ = 6.582, p = 0.086). Post-hoc tests on DR showed a significant difference in central tendency between AD (M = 0.361, SD = 0.360) and both HC (M = 0.801, SD = 0.337) (U = 38, p = 0.002) and SCD (M = 0.832, SD = 0.230) (U = 25, p = 0.001). All other comparisons were not significant (HC vs. SCD: U = 122.5, p = 0.627; HC vs. MCI: U = 109, p = 0.221, M MCI = 0.660, SD = 0.360; SCD vs. MCI: U = 97.5, p = 0.165; MCI vs. AD: U = 59.5, p = 0.033).

### Retrospective duration estimation

**Figure 6:**
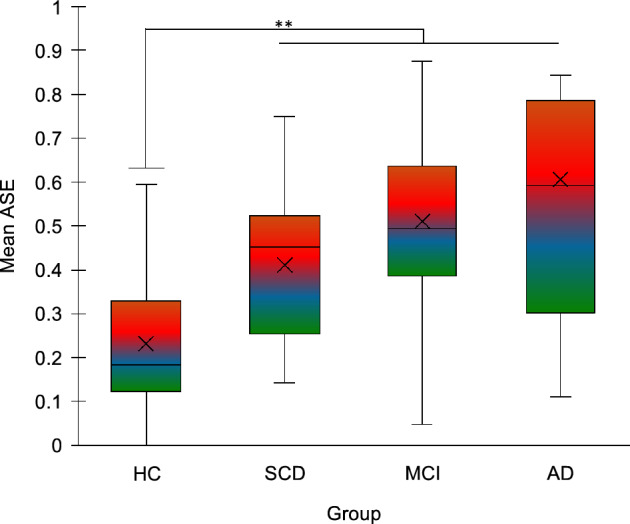
Results of the retrospective duration estimation task. Mean absolute standardized error (ASE) is plotted according to group. HC = healthy controls; SCD = Subjective Cognitive Decline; MCI = Mild Cognitive Impairment; AD = Alzheimer’s Disease. **p < 0.01. Error bars represent standard errors.

ASE was significantly different between groups (H_3_ = 16.255, p = 0.001), being lower in HC (M = 0.232, SD = 0.179) compared to the all the other groups (AD: M = 0.607, SD = 0.398, U = 39.5, p = 0.003; MCI: M = 0.510, SD = 0.218, U = 46, p = 0.001; SCD: M = 0.411, SD = 0.168, U = 60, p = 0.006). Other comparisons were not significant (Fig. 6).

### Prospective duration estimation in the minute range

**Figure 7:**
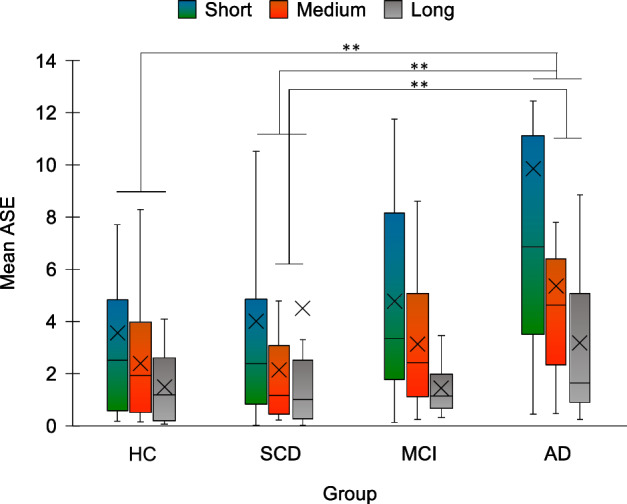
Mean absolute standardized error (ASE) of duration estimation in the minute range is plotted for each group and range of duration. HC = healthy controls; SCD = Subjective Cognitive Decline; MCI = Mild Cognitive Impairment; AD = Alzheimer’s Disease; Short = short duration range; Medium = medium duration range; Long = long duration range. **p < 0.01. Error bars represent standard errors.

ASE differed significantly according to Duration range (χ2_2_ = 76.097, *p* < 0.001). All post-hoc comparisons were significant, showing that ASE decreased with increasing duration (short: M = 5.303, SD = 0.278; medium: M = 3.143, SD = 3.354; long: M = 2.608, SD = 0.883, all *p* < 0.001). ASE also differed significantly between groups (H_3_ = 7.857, p = 0.049); however, no comparison survived Bonferroni’s correction. Differences between ASE in the short vs. long range (H_3_ = 11.57, p = 0.009) and in the medium vs. long range (H_3_ = 9.724, p = 0.021) were different between groups. Concerning the ASE difference between the short and the long range, decomposing the interaction showed a significant difference between AD (short: M = 9.859, SD = 10.557; long: M = 3.166, SD = 2.679) and both HC (short: M = 3.554, SD = 3.574; long: M = 1.493, SD = 1.406) and SCD (short: M = 3.997, SD = 4.775; long: M = 4.498, SD = 13.446), with the greater difference in AD compared to the other groups (AD vs. HC: U = 42, p = 0.004; AD vs. SCD: U = 40, p = 0.005). For the difference between the medium and long range, there was a significant difference between AD (medium: M = 5.349, SD = 5.051) and SCD (medium: M = 2.150, SD = 2.765) (U = 41, p = 0.006) (Fig. 7).

### Estimation of the date of public events

**Figure 8:**
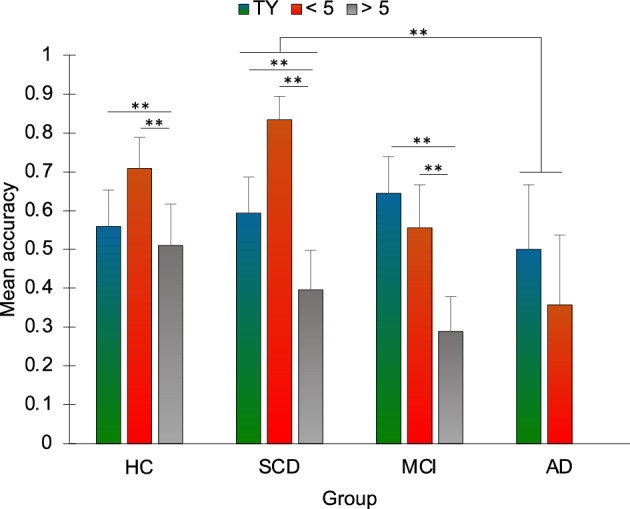
Mean accuracy in the estimation of the date of public events is plotted according to group and time period. HC = healthy controls; SCD = Subjective Cognitive Decline; MCI = Mild Cognitive Impairment; AD = Alzheimer’s Disease; TY: this year time period; < 5: within the last 5 years time period; > 5: more than 5 years ago time period. Bars represent standard errors. **p < 0.01. Error bars represent standard errors. Please note that the rightmost column is missing since, when considering only items for which participants reported to had known about the corresponding events, no correct response was provided in the AD group for the > 5 period.

Accuracy in estimating the date of public events differed significantly across periods (χ2 = 14.556, p = 0.001). Post-hoc comparisons showed that accuracy was significantly different between this year (TY) and more than 5 years ago (> 5) (TY: M = 0.580, SD = 0.396; > 5: M = 0.351, SD = 0.400; Z =—3.025, p = 0.002) and between the last 5 years (< 5) (M = 0.657, SD = 0.381) and > 5 (Z = -3.802, *p* < 0.001). Accuracy also differed between groups (H_3_ = 8.5, p = 0.037), with a significant difference between AD (M = 0.303, SD = 0.268) and SCD (M = 0.619, SD = 0.196) (U = 28.5, p = 0.006). Differences in accuracy between the levels of the Time period factors were not significantly different between groups (TY vs. < 5: H_3_ = 4.738, p = 0.192; TY vs. > 5: H_3_ = 6.364, p = 0.095; < 5 vs. > 5: H_3_ = 2.423, p = 0.489) (Fig. 8).

### Backward and forward telescoping

**Figure 9:**
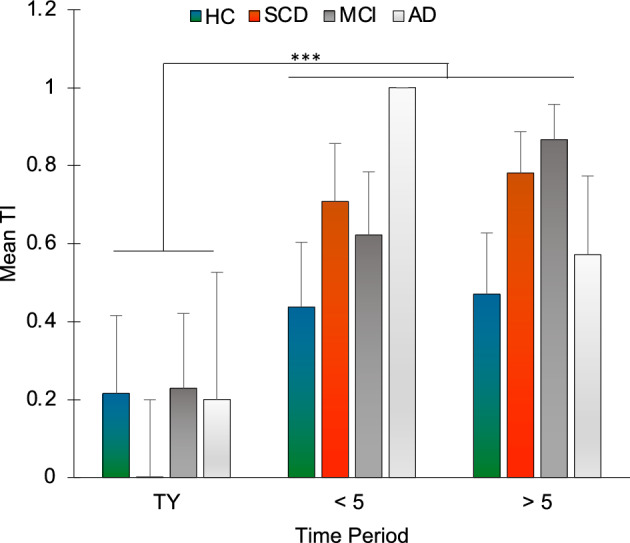
Mean telescoping index (TI) is plotted according to group and time period. HC = healthy controls; SCD = Subjective Cognitive Decline; MCI = Mild Cognitive Impairment; AD = Alzheimer’s Disease; TY: this year time period; < 5: within the last 5 years time period; > 5: more than 5 years ago time period. Bars represent standard errors. ***p < 0.001. Error bars represent standard errors.

The telescoping index (TI), assessing the tendency to attribute events to earlier or later dates, differed significantly according to Time period (χ2 = 16.211, *p* < 0.001), specifically between TY and < 5 (Z = -3.668, *p* < 0.001) and between TY and > 5 (Z = -3.457, p = 0.001), with lower TI in the TY period (TY: M = 0.158, SD = 0.824; < 5: M = 0.642, SD = 0.599; > 5: M = 0.682, SD = 0.515). There was no significant difference between groups (H_3_ = 0.897, p = 0.826) and differences between TI in the different levels of Time period also did not show differences according to Group (TY vs. < 5: H_3_ = 2.576, p = 0.462; TY vs. > 5: H_3_ = 4.457, p = 0.216; < 5 vs. > 5: H_3_ = 2.28, p = 0.516) (Fig. 9).

### Subjective experience of time

**Figure 10:**
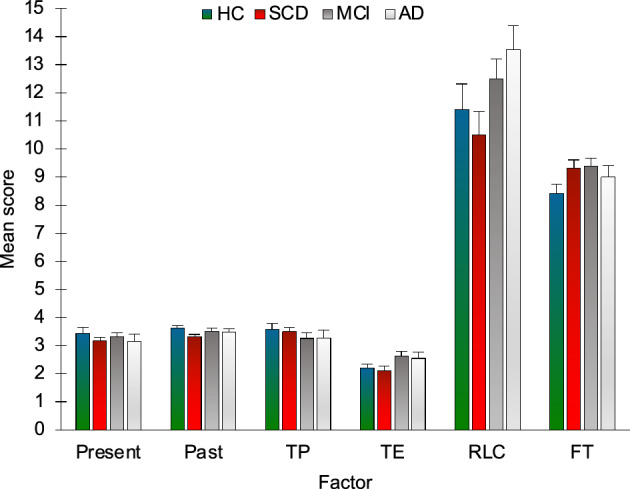
Scores on the questionnaire assessing the Subjective experience of time according to group and factor. HC = healthy controls; SCD = Subjective Cognitive Decline; MCI = Mild Cognitive Impairment; AD = Alzheimer’s Disease; Present = Personal experience of present; Past = Personal experience of past; TP = Time pressure; TE = Time expansion; RLC = Experience of recent life changes; FT = Forward telescoping. Bars represent standard errors.

No significant difference between groups was found for the factors Personal experience of present (H_3_ = 1.481, p = 0.687), Personal experience of past (H_3_ = 5.87, p = 0.118), Time pressure (H_3_ = 1.251, p = 0.741), Time expansion (H_3_ = 4.837, p = 0.184), Experience of recent life changes (H_3_ = 6.808, p = 0.078) and Forward telescoping (H_3_ = 5.389, p = 0.145) (Fig. 10).

A general overview of the main results of group comparisons is reported in Table 2.

**Table 2:** Overview of main findings. For each dimension of temporal processing assessed, duration range and a summary of patterns observed along the AD continuum are reported.

Time processing dimension	Range	Main findings
Internally-based temporal learning (IBL)	Seconds	No difference between groups
Externally-cued temporal learning (ECL)	Seconds	Impaired in AD patients; especially good performance in SCD individuals
Prospective duration estimation	Seconds	Impaired in AD patients
Prospective duration production	Seconds	Impaired in AD patients
Prospective duration reproduction	Seconds	Impaired in AD patients
Prospective duration estimation	Minutes	Lower performance in AD patients for the short and medium compared with the long range
Retrospective duration estimation	Minutes-hours	Lower performance in all groups compared to HC
Estimation of the date of public events	Months-years	Impaired in AD patients
Backward/forward telescoping	Months-years	No difference between groups
Subjective experience of time	Hours-decades	No difference between groups

### Correlation with neuropsychological tests

Since this was the first study systematically assessing different dimensions of time and duration processing along the continuum of AD, associations were explored between the experimental tasks and neuropsychological tests scores. Results are reported in Table 3. Significant negative correlations were highlighted between ASE in the ECL condition of the Bouncy ball task and measures assessing both short- and long-term memory, as well as attention, switching and executive functions. Similar patterns of correlations with performance on standard neuropsychological tests were also observed for ASE in the DR task and in prospective duration estimation in the range of minutes.

**Table 3:** Correlation between performance on the experimental tasks and scores on neuropsychological tests.

	MMSE	RAVLTI	RAVLTD	BSRTI	BSRTD	DST	CBTT	ROCFC	ROCFI	ROCFD	VS	TMTA	TMTB	PVFT	SVFT	BNT	FAB
IBL	− 0.176	− 0.148	− 0.021	0.008	0.074	− 0.105	− 0.06	− 0.126	− 0.168	− 0.114	− 0.192	0.126	0.197	− 0.179	− 0.052	0.008	− 0.056
ECL	**− 0.579**	**− 0.633**	**− 0.639**	**− 0.463**	**− 0.571**	**− 0.466**	**− 0.539**	− 0.467	**− 0.562**	**− 0.599**	− 0.358	**0.506**	**0.586**	− 0.449	**− 0.59**	− 0.45	**− 0.574**
DE	− 0.301	− 0.43	− 0.345	− 0.132	− 0.187	− 0.42	− 0.241	− 0.279	− 0.365	− 0.375	− 0.327	0.39	**0.743**	− 0.368	− 0.377	− 0.13	− 0.457
DP	− 0.322	− 0.376	− 0.422	− 0.149	− 0.375	− 0.222	− 0.26	− 0.404	**− 0.487**	**− 0.508**	− 0.285	**0.481**	0.429	− 0.213	− 0.413	− 0.274	− 0.395
DR	**− 0.455**	**− 0.649**	**− 0.605**	− 0.34	− 0.418	**− 0.602**	**− 0.476**	− 0.346	**− 0.551**	**− 0.577**	− 0.384	0.446	**0.738**	− 0.416	**− 0.552**	− 0.312	**− 0.607**
RDE	− 0.363	− 0.101	− 0.13	− 0.117	− 0.267	− 0.159	− 0.19	− 0.266	− 0.386	− 0.339	− 0.129	0.225	0.042	− 0.14	− 0.202	− 0.264	− 0.294
TEMS	**− 0.425**	− 0.346	− 0.322	− 0.192	− 0.352	− 0.401	**− 0.48**	− 0.318	**− 0.494**	**− 0.538**	− 0.29	0.249	0.434	− 0.292	− 0.398	− 0.273	**− 0.547**
TEMM	**− 0.485**	**− 0.463**	**− 0.461**	− 0.257	− 0.408	− 0.428	**− 0.498**	− 0.321	**− 0.507**	**− 0.616**	− 0.312	0.281	0.484	− 0.295	**− 0.496**	− 0.256	**− 0.598**
TEML	**− 0.409**	− 0.326	− 0.303	− 0.145	− 0.325	− 0.354	− 0.358	− 0.268	− 0.434	**− 0.479**	− 0.261	0.316	0.429	− 0.257	− 0.358	− 0.273	− 0.383
PEDTY	− 0.073	0.109	− 0.097	− 0.029	− 0.14	0.422	0.096	0.255	0.095	0.035	0.203	− 0.072	− 0.161	0.261	0.046	− 0.042	0.208
PED < 5	0.26	0.406	0.421	0.248	0.284	0.102	0.293	0.183	0.319	0.391	0.34	− 0.231	− 0.286	0.059	0.316	0.198	0.386
PED > 5	0.108	0.428	0.367	0.082	0.183	0.116	0.271	0.19	0.26	0.334	0.258	− 0.266	− 0.075	− 0.031	0.15	0.142	0.283
TITY	− 0.081	0.004	− 0.192	− 0.174	− 0.236	0.24	− 0.052	0.172	− 0.065	− 0.157	0.105	− 0.05	− 0.121	0.216	− 0.051	− 0.115	0.061
TI < 5	− 0.073	0.111	0.018	− 0.063	− 0.168	0.312	0.105	0.055	0.167	0.036	− 0.026	0.096	− 0.159	0.147	− 0.024	− 0.162	0.001
TI > 5	− 0.036	0.045	− 0.049	0.082	0.029	0.203	0.07	0.173	− 0.03	− 0.042	0.195	− 0.075	0.024	0.335	0.261	0.273	0.118
Pres	0.126	0.007	0.158	− 0.048	0.233	− 0.094	0.081	0.172	0.268	0.257	0.074	− 0.007	0.127	0.22	0.205	0.152	0.051
Past	− 0.028	0.059	− 0.04	− 0.312	− 0.09	0.135	0.021	− 0.097	− 0.006	0.073	− 0.084	0.186	0.087	0	− 0.09	− 0.137	0.005
TP	0.214	− 0.088	0.073	0.358	0.215	− 0.052	0.208	0.13	0.15	0.096	0.055	− 0.034	− 0.041	0.155	0.123	− 0.007	0.2
TE	**− 0.447**	− 0.227	− 0.275	− 0.231	− 0.39	− 0.117	− 0.212	− 0.365	− 0.205	− 0.248	− 0.066	0.251	0.025	− 0.143	− 0.281	− 0.088	− 0.121
RLC	− 0.299	− 0.398	− 0.391	− 0.271	− 0.311	− 0.274	− 0.26	− 0.398	− 0.291	− 0.316	− 0.394	0.328	0.172	− 0.316	− 0.379	− 0.249	− 0.353
FT	− 0.135	0.034	− 0.025	0.033	0.02	0.186	0.202	0.089	0.084	0.08	0.078	− 0.036	− 0.082	0.138	− 0.021	0.234	0.111

Two-tailed Spearman’s correlation coefficients are reported. Correlations surviving a threshold of *p* < 0.0013 are reported in bold. IBL = Bouncy ball task, Internally-Based Learning (ASE); ECL = Bouncy ball task, Externally-Cued Learning (ASE); DE = Duration estimation task (ASE); DP = Duration production task (ASE); DR = Duration reproduction task (ASE); RDE = Retrospective duration estimation (ASE); TEMS = Time estimation in the range of minutes, short range (ASE); TEMM = Time estimation in the range of minutes, medium range (ASE); TEML = Time estimation in the range of minutes, long range (ASE); PEDTY: Estimation of the date of public events, this year period (accuracy); PED < 5: Estimation of the date of public events, within the last 5 years period (accuracy); PED > 5: Estimation of the date of public events, more than 5 years ago period (accuracy); TITY: Telescoping index, this year period; TI < 5: Telescoping index, within the last 5 years period; TI > 5: Telescoping index, more than 5 years ago period; Pres = Experience of time in the present; Past = Experience of time in the past; TP = Time pressure; TE = Time expansion; RLC = Experience of recent life changes; FT = Forward telescoping; MMSE = Mini Mental State Examination; RAVLTI = Rey Auditory Verbal Learning Test, immediate recall; RAVLTD = Rey Auditory Verbal Learning Test, delayed recall; BSRTI = Babcock Story Recall test, immediate recall; BSRTD = Babcock Story Recall test, delayed recall; DST = Digit Span Test; CBTT = Corsi Block-Tapping Test; ROCFC = Rey-Osterrieth Complex Figure Test, copy; ROCFI = Rey-Osterrieth Complex Figure Test, immediate recall; ROCFD = Rey-Osterrieth Complex Figure Test, delayed recall; VS = Visual Search; TMTA = Trail Making Test A; TMTB = Trail Making Test B; PVFT = Phonemic Verbal Fluency Test; SVFT = Semantic Verbal Fluency Test; BNT = Boston Naming Test; FAB = Frontal Assessment Battery.

## Discussion

Here we performed the first systematic investigation of different levels of time processing along the continuum between healthy and pathological aging. Healthy elderly and individuals with SCD, MCI and AD performed a battery assessing prospective and retrospective time processing and the subjective experience of time. Based on evidence that implicit timing may show specific associations with age and cognitive decline^25,26^, our investigation included a novel task assessing implicit temporal learning (the Bouncy ball task) in an internally-based (IBL) and an externally-cued (ECL) condition^27–30,42^. Correlations were also assessed between time processing tasks and neuropsychological tests scores.

### Internally-based (IBL) and externally-cued (ECL) temporal learning

MCI and HC performed similarly in the two conditions of the Bouncy ball task. AD patients, instead, performed significantly worse in ECL than in IBL. Only one previous study has assessed the impact of cognitive decline on implicit timing, reporting a relation between lower MMSE scores and less efficient implicit time processing^26^. Our results further suggest that cognitive decline does not affect implicit time processing in tasks requiring internally-based timing (IBT), that is, the development of an internal referent of duration despite variations in environmental features^27^. IBT has shown to be is supported by a striato-thalamo-cortical network, involving prefrontal, motor and premotor regions, and the insula^27,29,30^. Gray matter loss along the trajectory of AD initially affects medial and inferior temporal lobes and the posterior cingulate/retrosplenial cortex, then progressing towards temporo-parietal association areas and the frontal lobes; motor, premotor regions and the insula, instead, are relatively spared^49–51^. The relative integrity of networks involved in IBT along the progression towards AD may thus possibly still support implicit learning of temporal information independently from the external environment, allowing MCI and AD patients to display unimpaired performance.

The ECL condition, instead, entailed learning to perform a response based on the regularity in an external sensory signal; in this condition, an outcome had to be determined based on co-occurrences of features within the observed stimuli (see Methods section). The tracking of statistics across experiences is a common element of different tasks requiring regularities integration in the range of minutes, including implicit and statistical learning paradigms (see^52^, for a review). Broad evidence exists for a key role of the hippocampus in this type of tasks^53–60^. Activation of the hippocampus has been also reported during a time-to-contact task, suggesting a role of this region in monitoring spatiotemporal regularities when the task requires to use implicit temporal information embedded in the regular temporal pattern of a stimulus^61^. Findings that AD performed worse in ECL than IBL may thus be explained considering the marked temporal lobes atrophy typically observed in these patients^51,62^. The link between the specific alterations of ECL in AD patients and hippocampal atrophy would be also in line with present findings that individuals with SCD showed an *opposite* pattern, i.e. *lower* absolute errors in ECL than IBL. SCD individuals often present worry and anxiety^63^, and show increased information sampling in uncertainty conditions, as well as increased insular-hippocampal intrinsic functional coupling^64^, suggesting that specific meta-cognitive operations may be at play in such individuals in uncertainty conditions. Although this interpretation is tentative at present, our results may reflect individuals with SCD having deployed more resources towards information sampling in the condition that was more hippocampal dependent – that is, ECL – resulting in higher performance. Supporting this possibility, individuals with SCD showed a pattern of performance similar to HC, but lower variability specifically in ECL (Fig. 3). Also, performance in ECL positively correlated with long-term memory, shifting and executive functioning tests scores. Such correlations were specific for ECL, suggesting that IBL may be more similar in nature to standard implicit timing tasks, that are assumed to impose low cognitive demands^25,26^. Results thus suggest that implicit time processing may be differently affected by dementia due to AD according to the specific task demands, in line with evidence that different procedures may support similar performance levels in timing tasks^65^ and with the involvement of multiple parallel brain systems in timing^66^.

### Prospective duration estimation, production and reproduction

AD patients showed higher absolute errors compared with the other groups in duration estimation, production and reproduction. MCI patients, instead, did not show altered performance. Findings are in line with evidence of reduced accuracy in prospective timing in AD^17,18^ (see^67^ for a review) but not MCI^10,18^. A significant difference in regression towards the mean was observed only between AD patients and HC and SCD, exclusively in duration reproduction. Central tendency measures in MCI fell between those of HC-SCD and those of AD patients, although no difference between MCI and other groups reached significance. While this result is not consistent with findings by Maaß and co-authors^23^, that showed stronger regression towards the mean in amnestic MCI patients compared with healthy elderlies, our duration reproduction paradigm differed from that by Maaß and colleagues^23^ both in the range of durations tested and in task structure. Moreover, it has been suggested that the relation between MCI and prospective time processing is moderated by several variables, including the stage of cognitive decline and the specific clinical features of the sample^10^. Thus, studies in larger samples may be necessary to assess the impact of different clinical features of MCI patients on performance in prospective timing tasks.

### Retrospective duration estimation

All groups, including individuals with SCD, showed higher errors than controls in retrospective duration estimation. Lower accuracy in AD patients compared to controls has been previously reported in retrospective time estimation^19^. Conversely, whereas a small-to-medium effect for an impairment was reported for AD and MCI patients in a recent meta-analysis^10^, other studies failed to find evidence for such alterations in MCI and SCD^21,68,69^. Pathological samples in these studies, however, included patients with vascular dementia and other neurological conditions, and control groups of healthy elderlies were substantially small or absent^68,69^. Retrospective time estimation has been suggested to rely on the retrieval of contextual changes associated with event sequences, that are encoded incidentally in medial temporal lobes as a part of spatio-temporal event context^8^. The encoding of temporal information in episodic memory has been proposed to be supported by the activity of hippocampal “time cells”, which firing patterns code for the evolution of temporal context during event sequences (^70^for a review). Accordingly, fMRI studies have shown that the hippocampus is sensitive to changes in the duration of empty intervals between stimuli of a sequence^71^ and accuracy in retrospectively discriminating durations predicts individual variations in the strength of intrinsic hippocampal connectivity^72^. Moreover, retrospective time estimates correlate with the ability to retrieve the context of experienced events^19^. Our results are consistent with this framework, showing alterations in retrospective duration processing along the continuum of AD, in which hippocampal atrophy is a key pathological hallmark. More importantly, such alterations can be identified also in SCD, that is, years before the onset of AD symptoms. Alterations in episodic components of autobiographical memory have been observed in APOE4 carriers, that show increased risk for cognitive decline related to AD^73,74^. From a theoretical point of view, our results thus support the possibility that the disruption of temporal coding – an integral component of episodic memory^75^—may contribute to the severe autobiographical amnesia in AD. From a clinical point of view, they open the door to the possibility that retrospective timing tasks may be used as a proxy of temporal context processing, allowing to identify early and subtle alterations in episodic memory, that are not routinely investigated in clinical settings.

### Prospective duration estimation in the minute range

Absolute errors in time estimation the minute range were inversely proportional to the duration range; this difference was accentuated in AD patients. In line with evidence on healthy young individuals^76,77^, all groups overestimated durations. Verbal time estimates have been suggested to be attracted towards verbal labels, with round outputs being more likely produced than intermediate ones^76–78^. Thus, while durations belonging to all the three ranges were overestimated, this “quantization” process may have particularly reduced estimation errors in judging “long” durations, which mean value was very close to 2 min. This effect may have been exaggerated in AD patients, in which cognitive estimation deficits^79,80^ may have particularly affected estimates for shorter durations, that were “more distant” from the 2 min label. Accordingly, absolute errors in this task correlated negatively with MMSE scores. Negative correlations were also found between absolute errors and scores on tests assessing long-term and working memory, consistently with proposals that judging intervals of multiple minutes involves both the retrieval of memory content of events occurred during the intervals, and attentional and working memory processes^76,77^.

### Estimation of the date of public events

Public events occurred during the last year and during the last five years were overall better collocated in time, compared to those occurred more than five years in the past. The collocation in time for events occurred more than five years in the past appeared to be particularly affected in AD patients, since, when considering only items for which they reported to knew about the corresponding event (see the Statistical analyses section), no correct response was provided in this group (Fig. 8). Higher forward telescoping effects (i.e., the tendency to underestimate the time passed since an event) were found, in all groups, for events occurred within the last five years and more than five years ago compared with events occurred within the last year. Whereas there are reports of more forward and backward telescoping for remote and recent events, respectively^14,81^, here we only highlighted the former effect. The task used in the present study differed from those employed by Janssen and colleagues^81^ and El Haj et al.^14^ in many respects, including stimuli and time periods. Moreover, given the small telescoping effect reported for very recent events^81^, this effect may have been overshadowed in the present study, since telescoping was calculated considering the time-period, rather than the absolute displacement of the event in days/months/years. Overall, our results are in line with evidence that telescoping effects for public events may be observed in AD, despite the severe impairment in dating such events^14,82,83^.

### Subjective experience of time

No difference between groups was found in scores on questionnaires assessing the experience of time^11–13^. MCI patients have been previously reported to experience time as passing more slowly than controls^21^. Although we did not observe this pattern, a negative correlation emerged between the feeling of time expansion and scores on MMSE, supporting previous observations of a relation between the experience of time passing more slowly and the reduction of the involvement in social and personal activity that is associated with the progression of cognitive decline^21^.

## Conclusions

This study has different limitations. Since biomarkers (e.g. CSF, plasma Abeta or tau, PET with amyloid tracers) were not collected, the diagnose of probable AD and MCI was performed according to NIAA clinical criteria^32,33^. Moreover, our battery did not include subsecond and/or purely motor or perceptual timing tasks. Our investigation focused on suprasecond timing since – given its relation with attention and working memory^84^—we expected it could be a more sensitive marker of impairment along the AD continuum. However, future studies should extend the present investigation, including the abovementioned measures, and replicating our findings in larger samples of patients. Moreover, considering previous evidence of deficits in mental time travel in patients with MCI^85^, as well early alterations in episodic autobiographical memory reported in individuals at risk of developing AD^73,74^, an investigation of mental time travel along the continuum of AD is also warranted to understand whether early impairments manifest also in time processing at longer timescales. This point is particularly important considering that duration processing appears to rely on different brain mechanisms according to the range of the durations tested: whereas timing of millisecond and second intervals has been shown to involve a network including the SMA, the basal ganglia, the insular cortex, the inferior and middle frontal gyrus as well as the intraparietal sulcus^86^, timing of durations at longer timescales (e.g. minutes and beyond) and memory for durations have been consistently associated with the involvement of the hippocampus^87^. In this respect, whereas alterations in temporal processing highlighted in this study in putatively hippocampal-dependent tasks support a role of medial temporal lobes in duration processing at specific timescales, findings that performance in prospective timing tasks was also altered in AD patients are in line with previous suggestions that, in this population, timing alterations in the second-range may be secondary to impairments in attention and working memory^88^.

Overall, present results provide new insights on alterations in temporal processing along the continuum of AD. First, implicit temporal learning appears to be less affected than explicit timing in pathological aging due to AD. Nonetheless, findings of a differential impairment of AD patients – as well as of a different performance in individuals with SCD – in internally-based and externally-cued implicit learning, highlight the importance to consider how specific task features may affect observed patterns of behavior even in implicit timing paradigms. Concerning explicit prospective timing tasks, our results confirm the presence of a substantial impairment in AD patients. However, as mentioned earlier, present findings do not allow to disentangle the issue of whether such alterations are due to impaired central temporal processing mechanisms, or to a more widespread attentional and working memory disfunction. Further research will be needed to assess more systematically the effect of such neuropsychological variables on different dimensions of temporal processing along the continuum of AD, including patients with MCI, in which evidence is still mixed. Present results further highlight alterations of time processing in AD patients at longer timescales, involving memory for durations and the collocation of events in time. On the one hand, this is consistent with a broad literature supporting the relation between episodic memory and time processing in tasks requiring the recovery of the temporal context of events^89^. On the other, findings that alterations in temporal context recovery as assessed by retrospective timing paradigms can also be observed in SCD contribute to a body of evidence increasingly showing subtle alterations in these individuals on measures of autobiographical memory specificity^90^. Finally, the subjective sense of the passage of time, spanning the timescale of multiple years and decades, was not found to be affected by the progression towards AD in the present study. While this finding may seem counterintuitive given the widespread temporal disorientation observed in AD patients^15^, different factors have been shown to contribute to explain differences in the experience of the passage of time, including time perspective and emotion regulation^12^, as well as event content^91^, highlighting the need of a more comprehensive assessment of factors underlying the subjective experience of time in healthy and pathological populations.

To conclude, a widespread alteration in time processing can be observed in AD, including prospective and retrospective timing, and the collocation of past events in time. MCI patients generally showed an intermediate pattern, with performances similar to those in AD, albeit less extreme. Finally, our findings show for the first time that specific changes in temporal processing may be observed also in SCD, that represents an early marker of future cognitive decline in a proportion of healthy individuals^92^. In this respect, retrospective duration processing appears as a particularly promising target for further research. Whereas the prospective estimation of time is critical to a wide range of cognitive processes, including motor control, distance and quantity judgments, as well as prospective memory^93,94^, it has been recently highlighted that many temporal judgments in real life involve the retrospective estimation of duration^95,96^. Future studies should thus aim to more deeply investigate alterations in retrospective timing in preclinical stages of AD, possibly using more structured tasks, involving a higher number of trials (e.g.^72^).

### Supplementary Information

Supplementary Information.

## Data Availability

The datasets generated during and/or analysed during the current study are available from the corresponding author on reasonable request.
