# Supplementary material for: Multidimensional assessment of time perception along the continuum of Alzheimer’s Disease and evidence of alterations in subjective cognitive decline
Source: Sci Rep. 2023 Dec 13;13:22117. doi: 10.1038/s41598-023-49222-x (PMC10719320; doi:10.1038/s41598-023-49222-x)
Supplement: Supplementary file 1 — Supplementary Information. [file 41598_2023_49222_MOESM1_ESM.pdf]

Supplementary information

Multidimensional assessment of time perception along the continuum of Alzheimer's Disease and evidence of alterations in Subjective Cognitive Decline

Alice Teghil[1,2*], Maddalena Boccia[1,2], Antonella Di Vita[3], Giulia Zazzaro[3], Micaela Sepe Monti[3], Alessandro Trebbastoni[3], Giuseppina Talarico[3], Alessandra Campanelli[3], Giuseppe Bruno[3], Cecilia Guariglia[1,2], Carlo de Lena[3], Fabrizia D'Antonio[2,3]

[1] Department of Psychology, Sapienza University of Rome, Rome, Italy
[2] Cognitive and Motor Rehabilitation and Neuroimaging Unit, IRCCS Fondazione Santa Lucia, Rome, Italy
[3] Department of Human Neuroscience, Sapienza University of Rome, Rome, Italy

*Corresponding author at:

Alice Teghil
Department of Psychology
"Sapienza" University of Rome
Via dei Marsi, 78, 00185 Rome, Italy

E-mail address: alice.teghil@uniroma1.it

Supplementary file S1

Items included in the questionnaire assessing time awareness and the subjective experience of time.

Rating scale:

1	2	3	4	5
Very slowly	Slowly	Neither fast nor slow	Fast	Very fast

Personal experience of present[1]

1. How fast does time usually pass for you?
2. How fast do you expect the next hour to pass?

Personal experience of past[1]

3. How fast did the previous week pass for you?
4. How fast did the previous month pass for you?
5. How fast did the previous year pass for you?
6. How fast did the previous 10 years pass for you?
7. How fast did your childhood (< 12 years) go by?
8. How fast did your youth (13-19 years) go by?
9. How fast did your adulthood between 20 and 29 years go by?
10. How fast did your adulthood between 30 and 39 years go by?

Rating scale:

1	2	3	4	5
Strongly disagree	Disagree	Neither agree nor disagree	Agree	Strongly agree

Time pressure[1]

11. I haven't enough time to complete my tasks.
12. I often feel time pressure.
13. I often haven't enough time to devote myself to important things.
14. I often think time is running out.
15. I have to establish my priorities, because I cannot do all the things I would like to do.

Time expansion[1]

16. My time seems empty.
17. I often think that time just does not want to pass.
18. I often feel bored.
19. I have a lot of time.
20. I often have spent my time without doing anything.

Experience of recent life changes[2]

21. The past two years have been a time filled with many new experiences (reverse scored).
22. In the past several years, my life has been quite routine.
23. When I think back over the past two years, few notable events come to mind.
24. There have been few notable changes in my life in the past year.

Forward telescoping[2]

25. When I try to remember the date of some event, I often come up with a time that is not as long ago as the true time.
26. When I think that something was just a few years ago, it often turns out that it happened long before that.
27. I often find that things actually occurred much longer agothan I thought (reverse scored).

[1] Items from Wittmann and Lehnhoff (2005).
[2] Items from Friedman and Janssen (2010).

Supplementary file S2

Analyses on ratio scores in prospective duration estimation, production and reproduction tasks

In order to assess differences in the direction of the errors between groups, we calculated the ratio of the estimated, produced and reproduced to the target duration in the four groups. Ratio scores were compared between groups for each task separately, performing three Kruskal-Wallis tests. When a significant difference was identified, paired comparisons were performed between groups using Mann-Whitney tests (alpha level set at 0.0083, applying Bonferroni's correction for multiple comparisons).

Results showed a significant difference in the ratio scores between groups in duration production (DP) (H_3 = 8.362, p = 0.039) but not in duration estimation (DE) (H_3 = 2.026, p = 0.567) or duration reproduction (DP) (H_3 = 7.126, p = 0.068). In post-hoc comparisons, no difference survived Bonferroni correction (HC vs. SCD: U = 119, p = 0.540; HC vs. MCI: U = 95, p = 0.088; HC vs. AD: U = 53, p = 0.016; SCD vs. MCI: U = 102, p = 0.221; SCD vs. AD: U = 59, p = 0.048; MCI vs. AD: U = 71, p = 0.098). Means and standard deviations for the four groups in the three tasks are reported below.

Task	Group	M	SD
**DE**	**HC**	1.470	0.792
	**SCD**	1.318	0.570
	**MCI**	1.475	0.625
	**AD**	2.039	2.089
**DP**	**HC**	1.002	0.436
	**SCD**	1.079	0.421
	**MCI**	1.210	0.294
	**AD**	1.444	0.478
**DR**	**HC**	1.002	0.242
	**SCD**	0.926	0.116
	**MCI**	1.042	0.266
	**AD**	1.152	0.260

Analyses on ratio scores in retrospective duration estimation

The ratio of the estimated to the target duration was calculated in the four groups for the retrospective duration estimation task. Ratio scores were compared between groups using a Kruskal-Wallis test. Paired comparisons were performed between groups using Mann-Whitney tests (alpha level set at 0.0083, Bonferroni's correction for multiple comparisons).

Ratio scores differed significantly between groups (H_3 = 9.842, p = 0.02). Post-hoc comparisons showed a significant difference between HC (M = 0.789, SD = 0.205) and MCI (M = 0.513, SD = 0.269) (U = 53, p = 0.003). All other comparisons did not survive correction for multiple comparisons (HC vs. SCD [M = 0.612, SD = 0.218]: U = 67, p = 0.013; HC vs. AD [M = 0.728, SD = 0.691]: U = 76.5, p = 0.155; SCD vs. MCI: U = 90, p = 0.152; SCD vs. AD: U = 85, p = 0.404; MCI vs. AD: U = 100.5, p = 0.878).

Analyses on ratio scores in prospective duration estimation in the minute range

To assess possible differences as a function of the duration range, independently from the group (i.e. testing the main effect of Duration range), a Friedman test was performed on the estimated-to-target-duration ratio scores with the factor Duration range (short, medium, long). A Kruskal-Wallis test was also performed on ratio scores to assess the main effect of Group (HC, SCD, MCI, AD), independently from the duration range. Finally, the interaction between factors Duration range and Group was also assessed adopting a non-parametric approach: we first calculated differences between mean ratio scores in each group for each pairing of levels of the Duration range factor (i.e. mean ratio score was calculated within each group for differences between the short and medium condition, the short and long condition, and the medium and long condition). Then, groups were compared on such differences using Kruskal-Wallis tests. Wherever Kruskal-Wallis tests showed that differences between the levels of the repeated-measures factor did indeed differ significantly between groups, groups' means were compared using Mann-Whitney tests (alpha was set at 0.0083, applying Bonferroni's correction).

Results showed that the ratio differed significantly according to Duration range ($\chi2_2$ = 75.903, $p < 0.001$). All post-hoc comparisons were significant, showing that the ratio decreased with increasing duration (short: M = 6.243, SD = 6.334; medium: M = 4.075, SD = 3.402; long: M = 3.540, SD = 6.973, all *p*s < 0.001). The ratio also differed significantly between groups (H_3 = 7.897, p = 0.048), however, no comparison survived Bonferroni's correction (HC [M = 3.401, SD = 2.432] vs. SCD [M = 4.482, SD = 6.774]: U = 134, p = 0.934; HC vs. MCI [M = 4.055, SD = 2.181]: U = 102, p = 0.221; HC vs. AD [M = 7.023, SD = 5.936]: U = 58, p = 0.028; SCD vs. MCI: U = 94, p = 0.200; SCD vs. AD: U = 50, p = 0.018; MCI vs. AD: U = 67, p = 0.105). Differences between ratios in the short vs. long range (H_3 = 11.214, p = 0.011) and in the medium vs. long range (H_3 = 10.791, p = 0.013) were different between groups, whereas that between the short and medium range was not (H_3 = 3.304, p = 0.347). Concerning the difference between the short and the long range, decomposing the interaction showed a significant difference only between HC (short: M = 4.475, SD = 3.607; long: M = 2.429, SD = 1.483) and AD (short: M = 10.770, SD = 10.599; long: M = 4.164, SD = 2.682) (U = 43, p = 0.005). For the difference between the medium and long range, there was a significant difference between SCD (medium: M = 3.056, SD = 2.838) and MCI (medium: M = 4.106, SD = 2.438) (U = 53, p = 0.005).

Supplementary Table S1

Stimulus parameters in the temporal learning task (Bouncy ball task).

Condition	Trial	Bouncing duration (ms) (1)	Bouncing duration (ms) (2)	Bounces N° (1)	Bounces N° (2)	FPS (1)	FPS (2)	FPS (test 1)	FPS (test 2)	Target duration (ms) (test 1)	Target duration (ms) (test 2)
IBL	1	3100	3100	3	6	15	30	50	40	3100	3100
	2	6350	6350	6	12	15	30	40	50	6350	6350
	3	3100	3100	3	6	15	30	50	40	3100	3100
	4	4700	4700	9	6	30	20	40	50	4700	4700
	5	6350	6350	12	6	30	15	40	50	6350	6350
	6	4700	4700	6	9	20	30	50	40	4700	4700
ECL	1	2133.33	4266.67	4	4	30	15	40	50	1600	1280
	2	4800	2400	9	9	30	60	50	40	2880	3600
	3	4800	3200	6	6	20	30	50	40	1920	2400
	4	2400	4800	9	9	60	30	50	40	2880	3600
	5	2133.33	4266.67	4	4	30	15	40	50	1600	1280
	6	4800	3200	6	6	20	30	40	50	2400	1920

Bouncing duration (1) and (2): the two total time intervals for which the bouncy ball stimulus bounced across the six repetitions in the learning phase; Bounces N° (1) and (2): the two alternating numbers of bounces of the stimulus across the six repetitions in the learning phase; FPS (frames per second) (1) and (2): the two alternating bouncing speeds of the stimulus across the six repetitions in the learning phase; FPS (test 1) and (test 2): the two alternating speeds of the stimulus in the two response moments of the test phase; Target duration (test 1) and (test 2): target duration in each of the two response moments of the test phase.

Supplementary Table S2
Detailed information on stimuli used in the Newscast task.

Version	Topic	Category	Duration (sec)	Duration range	Time period	Date
1	Paolo Villaggio's death	EN	121	L	TY	03/07/17
1	Emmanuel Macron's election	P	204	L	TY	08/05/17
1	Rigopiano avalanche	NS	78	M	TY	19/01/17
1	Brexit announcement	P	120	L	< 5	25/06/16
1	Charlie Hebdo shooting	NS	102	M	< 5	07/01/15
1	Academy Award to "The Great Beauty"	EN	79	M	< 5	03/03/14
1	Prince William and Kate Middleton's wedding	NS	50	S	> 5	28/04/11
1	Sanremo Festival opening night	EN	56	S	> 5	18/02/09
1	John McCain designed as George W. Bush's successor	P	48	S	> 5	03/09/08
2	Morandi Bridge collapse	NS	78	M	TY	16/08/18
2	Italian elections	P	124	L	TY	23/01/18
2	Catherine Deneuve/Mee Too movement polemic	EN	58	S	TY	10/01/18
2	Paolo Villaggio's death	EN	121	L	< 5	03/07/17
2	Brexit announcement	P	95	M	< 5	25/06/16
2	Charlie Hebdo shooting	NS	43	S	< 5	07/01/15
2	Academy Award to "The Great Beauty"	EN	79	M	> 5	03/03/14
2	Prince William and Kate Middleton's wedding	NS	118	L	> 5	28/04/11
2	John McCain designed as George W. Bush's successor	P	48	S	> 5	03/09/08

For each clip, the topic, general category, duration, duration range, specific time period and airing date is reported. EN = entertainment news; P = politics; NS = news stories; L = long; M = medium; S = short; TY = this year; <5 = within the last 5 years; > 5 = more than 5 years ago.
